# Persistence on HIV preexposure prophylaxis medication over a 2‐year period among a national sample of 7148 PrEP users, United States, 2015 to 2017

**DOI:** 10.1002/jia2.25252

**Published:** 2019-02-18

**Authors:** Kelsey C Coy, Ronald J Hazen, Heather S Kirkham, Ambrose Delpino, Aaron J Siegler

**Affiliations:** ^1^ Department of Epidemiology Rollins School of Public Health Emory University Atlanta GA USA; ^2^ Walgreen Co. Deerfield IL USA; ^3^ Department of Behavioral Sciences and Health Education Rollins School of Public Health Emory University Atlanta GA USA

**Keywords:** PrEP, retention, prevention, medication persistence, preventative medicine, HIV

## Abstract

**Introduction:**

Persistence on preexposure prophylaxis for HIV prevention (PrEP) medication has rarely been reported for periods greater than one year, or in real‐world settings. This study used pharmacy fill records for PrEP users from a national chain pharmacy to describe persistence on PrEP medication over a two‐year period, and to explore correlates with PrEP medication persistence in a real‐world setting.

**Methods:**

We analysed de‐identified pharmacy fill records of 7148 eligible individuals who initiated PrEP in 2015 at a national chain pharmacy. A standard algorithm was employed to identify TDF‐FTC use for PrEP indication. We considered three time periods for persistence, defined as maintaining refills in PrEP care: year 1 (zero to twelve months), year 2 (thirteen to twenty‐four months) and initiation to year 2 (zero to twenty‐four months). Individuals with 16 or more days of TDF‐FTC PrEP dispensed in a 1‐month period for at least three‐quarters of a given time period (e.g. nine of twelve months or eighteen of twenty‐four months) were classified as persistent on PrEP medication for the period.

**Results:**

Persistence was 56% in year 1, 63% in year 2 and 41% from initiation to year 2. Individuals aged 18 to 24 had the lowest persistence, with 29% from initiation to year 2. Men had higher persistence than women, with 42% compared to 20% persistent from initiation to year 2. Individuals with commercial insurance and individuals who utilized a community‐based specialty pharmacy from the national chain also had higher persistence. Male gender, age >18 to 24 years, average monthly copay of $20 or less, commercial insurance, and utilization of a community‐based specialty pharmacy were positively associated in adjusted models with persistence in year 1 and from initiation to year 2; the same correlates, with the exception of utilization of a community‐based specialty pharmacy, were associated with higher persistence in year 2.

**Conclusions:**

We found substantial non‐persistence on PrEP medication in both year 1 and year 2. Across the entire 2‐year period, only two out of every five users persisted on PrEP. Demographic, financial and pharmacy factors were associated with persistence. Further research is needed to explore how social, structural or individual factors may undermine or enhance persistence on PrEP, and to develop interventions to assist persistence on PrEP.

## 1 Introduction

Preexposure prophylaxis (PrEP) for HIV prevention with daily oral use of tenofovir disoproxil fumarate and emtricitabine (TDF‐FTC) is well‐tolerated and highly effective 1, 2, 3, 4, 5, 6, 7, 8. PrEP is still relatively new to clinical settings; the United States Food and Drug Administration approved this combination for HIV prevention in 2012, and the Centers for Disease Control and Prevention published clinical practice guidelines in 2014 (updated in 2017), with interim guidelines first published in 2011 9, 10. Despite ample evidence of effectiveness in clinical trial settings, there is less known about PrEP use, adherence and persistence in real‐world settings.

Definitions of persistence on PrEP medication are often based on maintaining all aspects of recommended care per CDC guidance for the duration of a study period: adequate adherence to medications, HIV and sexually transmitted infection (STI) testing every three months, and creatinine testing every six months 10, 11. Such a definition is useful for clinical trials, yet is difficult to operationalize with currently available, population‐based datasets. Administrative datasets, such as those from commercial aggregators, health insurers or pharmacies, allow for population‐based assessments of persistence in multiple disease states by considering the length of therapy prior to discontinuation 11, 12, 13, 14, 15. Pharmacy refill data have been validated as an approach to assess adherence and persistence on medication 16, 17.

To date, a number of clinical trials and demonstration projects have assessed PrEP efficacy and adherence for periods of time up to two years, but there have been few clinical trials or clinical practice studies that analysed PrEP persistence over periods greater than six months. Evidence from clinical trials indicates that if participants are retained past an initial period of PrEP medication use then they are more likely to remain on PrEP. Longitudinal analyses in the Partners PrEP clinical trial demonstrated that PrEP medication use remained high throughout the 3‐year study period among the 70% of participants who had high TDF‐FTC levels in the first month; in contrast, the 30% of participants with no‐ or low‐detectable drug levels in the first month had low or inconsistent PrEP use throughout the study 18. The open‐label randomized PROUD trial found sufficient medication was filled for 88% of the total 2‐year follow‐up time 7. Clinical practice evidence indicates substantial non‐persistence on PrEP over time, which is associated with demographic and financial factors. In one clinical practice study of men who have sex with men (MSM) in three mid‐sized U.S. cities, 73% were persistent at three months and 60% were persistent at six months; furthermore, insurance status and medication costs were not found to be significant barriers to persistence 19. A follow‐up survey of MSM in Miami and San Francisco who had completed the United States PrEP Demonstration Project four to six months prior found that only 40% had taken PrEP since the study's completion, despite 92% having reported interest in continuing PrEP. Cost and lack of insurance were the greatest perceived barriers to accessing PrEP; additionally, being older than 18 to 25, having health insurance, and being willing to pay for PrEP were positively associated with accessing PrEP 20.

The objectives of this study are as follows: (1) to describe persistence on PrEP medication over a 2‐year period using pharmacy refill data from a national pharmacy chain and (2) to explore correlates with persistence on PrEP.

## 2 Methods

We analysed deidentified data from a simple random sample of all PrEP users who initiated PrEP in 2015 at one national chain pharmacy. TDF‐FTC prescriptions were identified as PrEP prescriptions, rather than use for HIV treatment or post‐exposure prophylaxis, if they met the following conditions: (1) at least a 60‐day supply of TDF‐FTC in 2015 and (2) antiretroviral monotherapy (e.g. no other antiretroviral prescriptions filled). Individuals aged <18 or who had a PrEP prescription in 2014 were not eligible for the analysis. For each eligible individual, data were collected for a 24‐month period after PrEP initiation. This sample constituted a substantial proportion of individuals prescribed TDF‐FTC for PrEP in the overall pharmacy dataset. Additionally, during the study period, the national chain pharmacy had locations in 49 states and 20 of the most populous cities.

Data for 7148 eligible individuals had information for the three outcomes of interest: persistence in year 1 (months 0 to 12), persistence in year 2 (months 13 to 24) and persistence from initiation to year 2 (months 0 to 24). Persistence was defined as having at least 16 days of PrEP medication filled per 30‐day period, starting at the index fill date, for at least three‐quarters of a period (nine months in a twelve‐month period or eighteen months in a twenty‐four‐month period). For this analysis, we defined a month as a 30‐day period from the initiation date, as opposed to calendar month. The periods included year 1, defined as months 0 to 12 after the index fill date; year 2, defined as months 13 to 24 after the index fill date; and initiation to year 2, defined as months 0 to 24 after the index fill date. Persons who were not persistent in year 1 were excluded from being persistent in year 2. We defined persistence thresholds of at least 16 days per month because dosing at a minimum level of four days per week has been shown to offer substantial protection 19, 21. An array was used to adjust for overlapping medication supply and potential stockpiling during the study period.

Variables included in the analysis were derived from administrative pharmacy claims: demographics (age category and gender), financial information (average copay per month and primary payer type), geographic information (driving distance in miles from individual's home address to nearest store and urban/rural status of pharmacy) and pharmacy type. Race/ethnicity, gender identity, sexual orientation and income are known areas of disparities in HIV transmission and in PrEP uptake; however, this dataset did not contain information on these key variables. Age was categorized as 18 to 24, 25 to 29, 30 to 39, 40 to 49 and 50 or older, and gender was categorized as men or women based on insurance information. Average copay per month was calculated as the average monthly out‐of‐pocket payment per individual over the length of time PrEP medication was filled. Monthly copay amount reflects only the amount paid by the individual after insurance, copay assistance or other programmes have been utilized. Primary payer was identified as the first payer billed for the majority of PrEP prescriptions during the period. Primary payer had the following categories: commercial (all commercial insurances), government (Medicare/Medicaid), or cash/other (primarily paying with cash, manufacturer's copay assistance programme, or other/unknown sources). In instances when two sources of payment were received, the primary payer variable prioritized commercial or government health insurance coverage over manufacturer and copay assistance (i.e. secondary coverage), and thus is not an accurate reflection of use of manufacturer copay and medication assistance programmes. When the manufacturer's copay assistance programme was used as the sole source of payment, it was categorized as cash/other. Copay assistance programmes are designed to support part of the cost of the medication, whereas medication assistance programmes provide full financial coverage of the medication for individuals with financial need. Driving distance was calculated using ArcGIS to calculate distance from the individual's home address to the nearest pharmacy in the national chain. Urban/rural status of the pharmacy nearest to the individual's address was categorized as urban, less dense urban, suburban and rural using the national pharmacy chain's proprietary algorithm that accounts for population density. For pharmacy type, we determined whether individuals utilized a community‐based specialty pharmacy or a traditional retail pharmacy from the national pharmacy chain. Community‐based specialty pharmacies have staff with additional training in HIV stigma and prevention, medication access, financial assistance coordination, treatment guidance and adherence support. Due to the nature of administrative data, demographic data points were missing for several records; however, we assumed data were missing at random and did not impact the results of the analysis.

For each of the three periods of persistence, we used bivariate and multivariable logistic regression models to assess predictors of persistence on PrEP medication. Variables were considered for inclusion in multivariable models based on significance at the 0.1 level in bivariate analysis. Variables included in final multivariable models were significant at the 0.05 level. Collinearity was evaluated for each model, and model fit statistics were calculated using Hosmer‐Lemeshow tests. All analyses were conducted using SAS Version 9.4 (SAS Institute). Data visualizations were conducted in Microsoft Excel 2016.

Individuals who used a pharmacy outside of this national chain during the study period could be misclassified as discontinuing PrEP. To explore this source of bias we conducted a sensitivity analysis, including only individuals who had filled a prescription (excluding PrEP) at the national pharmacy chain of interest *after* the 24‐month study period had ended. Descriptive and modelling analyses described above were repeated in their entirety on this subset, which included 5837 individuals.

This research was approved by Quorum IRB (#30978/1) with waivers of informed consent and HIPAA authorization. Only de‐identified observational data were shared with Emory University.

## 3 Results

### 3.1 Demographics

Among 7148 persons who initiated PrEP in 2015 at a large national chain pharmacy, 97% were men and 3% were women (Table 1). The plurality (35%) were age 30 to 39, 22% were 25 to 29, 20% were 40 to 49, 12% were 50 or older, and 11% were 18 to 24. Over three‐quarters (77%) had a monthly PrEP copay of $20 or less. The majority had commercial insurance (80%), 15% had government insurance, and 5% had either cash, manufacturer's copay assistance programme, or other/unknown denoted as their primary payer. A minority utilized a community‐based specialty pharmacy from the national chain (15%). Nearly three‐quarters of individuals had a pharmacy within one mile of their home and most resided in urban (63%) or suburban (32%) locations, with only 5% rural.

**Table 1:** Demographic characteristics of individuals who initiated HIV PreExposure prophylaxis in the United States, 2015 at initiation

	n (%)
Total sample	7148
Age	
18 to 24	784 (11%)
25 to 29	1552 (22%)
30 to 39	2521 (35%)
40 to 49	1432 (20%)
50+	855 (12%)
Gender	
Men	6900 (97%)
Women	244 (3%)
Monthly average copay	
$20 or less	5531 (77%)
More than $20	1614 (23%)
Mean (SD)	20 (78)
Payer (primary during entire period)	
Commercial	5699 (80%)
Government	1097 (15%)
Cash/other	352 (5%)
Pharmacy type	
Community‐based specialty pharmacy	1057 (15%)
Traditional retail pharmacy	6091 (85%)
Distance to pharmacy from home (miles)	
0 to <1 miles	5293 (74%)
1 to <2 miles	1235 (17%)
2+ miles	620 (9%)
Mean (SD)	1 (3)
Urban/rural status	
Urban	3093 (43%)
Less dense urban	1458 (20%)
Suburban	2257 (32%)
Rural	340 (5%)

Primary payer reflects the source of payment used most frequently in the study period. Some data points are missing for up to four individuals.

### 3.2 Persistence

**Figure 1:**
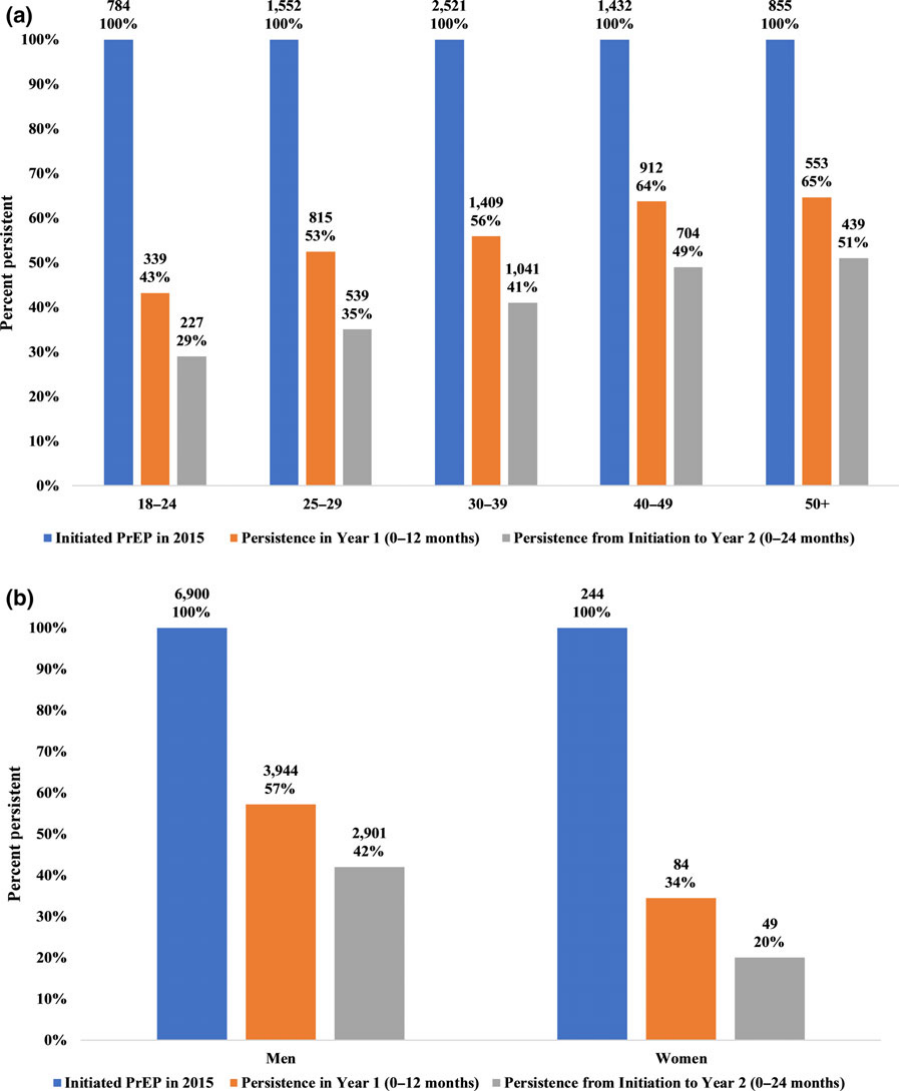
(**a)** Persistence on PrEP medication over time by age group. **(b)** Persistence on PrEP medication over time by gender. To be considered persistent, individuals must have had 16 days of medication available per calendar month for three‐quarters of months in a period. Some data points are missing for up to four individuals.

In year 1, 56% (4030/7148) were classified as persistent on PrEP medication (Table 2). Among individuals that were persistent in year 1, 63% (2521/4030) were persistent in year 2. From initiation to year 2, 41% (2951/7148) were persistent. Overall, persistence was only slightly higher for year 2 (63%) than for year 1 (56%). The two variables with the largest range in persistence outcomes from initiation to year 2 were gender and age (see Table 2 and Figure 1). No age group or gender had greater than 54% persistence from initiation to year 2. The lowest proportion persistent on medication in year 1, year 2, and from initiation to year 2 were women and age group 18 to 24, with women having 34%, 49% and 20% persistence, and the 18 to 24 age group having 43%, 54% and 29% persistence respectively. See Table S1 for full results.

**Table 2:** Persistence on PrEP medication in year 1 (zero to twelve months), year 2 (twelve to twenty‐four months), and initiation to year 2 (zero to twenty‐four months) among individuals who initiated PrEP in the United States, 2015 for selected variables

	PrEP initiation	Persistence in year 1 (zero to twelve months)		Persistence in year 2 (thirteen to twenty‐four months)		Persistence from initiation to year 2 (zero to twenty‐four months)	
	n	n	Percent persistent	n	Percent persistent	n	Percent persistent
All	7148	4030	56%	2521	63%	2951	41%
Age							
18 to 24	784	339	43%	183	54%	227	29%
25 to 29	1552	815	53%	452	55%	539	35%
30 to 39	2521	1409	56%	872	62%	1041	41%
40 to 49	1432	912	64%	621	68%	704	49%
50+	855	553	65%	392	71%	439	51%
Gender							
Men	6900	3944	57%	2479	63%	2901	42%
Women	244	84	34%	41	49%	49	20%

To be considered persistent, individuals must have had 16 days of medication available per calendar month for three‐quarters of months in each period. Only individuals persistent at one year of follow‐up (months 0 to 12) were eligible to be considered persistent at two years (months 13 to 24). Variables in this table are significantly associated with PrEP discontinuation; see Table 3 for more detail. Some data points are missing for up to four individuals.

An analysis to explore possible re‐entry into PrEP medication found that only 12% (369/3118) of those who were classified as not persistent in year 1 (months 0 to 12) would be classified as persistent in their second year of follow‐up (months 13 to 24) (data not shown).

### 3.3 Predictors of refill persistence

In multivariable analysis, male gender, being older than 18 to 24 years, having a copay of $20 or less, having commercial insurance, and attending a community‐based specialty pharmacy from the national chain were associated with persistence in year 1 of follow‐up (zero to twelve months) (Table 3). Excluding attending a community‐based specialty pharmacy, these variables were also associated with persistence in year 2 (13 to 24 months). The same variables were associated with persistence for the initiation to year 2 period (zero to twenty‐four months) as for the year 1 period; see Table 3 for odds ratios. No models had evidence of poor fit or collinearity.

**Table 3:** Factors associated with persistence on PrEP medication in year 1 (zero to twelve months), year 2 (twelve to twenty‐four months), and initiation to year 2 (zero to twenty‐four months) among individuals who initiated PrEP in the United States, 2015

	Persistence in year 1 (zero to twelve months) n=7141		Persistence in year 2 (thirteen to twenty‐four months) n=4030		Persistence from initiation to year 2 (zero to twenty‐four months) n=7141	
	Bivariate OR (95% CI)	Multivariable aOR (95% CI)	Bivariate OR (95% CI)	Multivariable aOR (95% CI)	Bivariate OR (95% CI)	Multivariable aOR (95% CI)
Age						
18 to 24	Ref	Ref	Ref	Ref	Ref	Ref
25 to 29	1.45 (1.22, 1.73)	1.43 (1.20, 1.71)	1.06 (0.82, 1.37)	1.06 (0.82, 1.37)	1.31 (1.08, 1.57)	1.28 (1.06, 1.54)
30 to 39	1.66 (1.42, 1.96)	1.66 (1.41, 1.96)	1.38 (1.09, 1.76)	1.39 (1.09, 1.76)	1.73 (1.45, 2.05)	1.71 (1.44, 2.04)
40 to 49	2.30 (1.93, 2.75)	2.37 (1.98, 2.84)	1.82 (1.41, 2.35)	1.87 (1.45, 2.42)	2.37 (1.97, 2.86)	2.43 (2.01, 2.94)
50+	2.40 (1.97, 2.93)	2.57 (2.10, 3.15)	2.08 (1.57, 2.75)	2.26 (1.70, 3.01)	2.59 (2.11, 3.18)	2.77 (2.25, 3.41)
Gender						
Women	Ref	Ref	Ref	Ref	Ref	Ref
Men	2.54 (1.94, 3.32)	2.25 (1.70, 2.97)	1.78 (1.15, 2.74)	1.61 (1.03, 2.52)	2.89 (2.10, 3.96)	2.46 (1.77, 3.41)
Monthly average copay						
$20 or less	Ref	Ref	Ref	Ref	Ref	Ref
More than $20	0.72 (0.64, 0.80)	0.63 (0.56, 0.71)	0.78 (0.67, 0.92)	0.68 (0.58, 0.81)	0.70 (0.63, 0.79)	0.61 (0.54, 0.69)
Payer (primary during entire period)						
Commercial	Ref	Ref	Ref	Ref	Ref	Ref
Government	0.59 (0.52, 0.68)	0.58 (0.50, 0.67)	0.62 (0.51, 0.75)	0.59 (0.48, 0.71)	0.52 (0.46, 0.60)	0.51 (0.44, 0.59)
Cash/other	0.56 (0.46, 0.70)	0.54 (0.44, 0.68)	0.72 (0.52, 0.99)	0.69 (0.50, 0.96)	0.62 (0.50, 0.78)	0.59 (0.47, 0.75)
Pharmacy type						
Traditional retail pharmacy	Ref	Ref	Ref	NS	Ref	Ref
Community‐based specialty pharmacy	1.43 (1.25, 1.63)	1.42 (1.24, 1.64)	1.18 (0.99, 1.40)		1.41 (1.23, 1.60)	1.41 (1.23, 1.61)
Distance to pharmacy from home (miles)	NS		NS		NS	
Urban/rural status	NS		NS		NS	

NS, not significant at the *p* < 0.05 level. To be considered persistent, individuals must have had 16 days of medication available per calendar month for three‐quarters of months in each period. Only individuals persistent at one year of follow‐up (months 0 to 12) were eligible to be considered persistent at two years (months 13 to 24). Primary payer reflects the source of payment used most frequently in the study period. Some data points are missing for up to four individuals.

### 3.4 Sensitivity analysis

A sensitivity analysis of a subset of 5837 individuals who had filled any prescription other than PrEP after their final PrEP fill after the follow‐up period of 24 months demonstrated few differences between the subset and the larger PrEP sample, indicating that switching pharmacies is likely not a substantial contributor to the rates of drop off from filling PrEP medication. See Tables S2 and S3 in the Supporting Information. In multivariable analysis, the same predictors were associated with persistence in year 1 as were associated with the same outcomes in the multivariable analysis on the larger sample; however, for persistence in year 2 and persistence from initiation to year 2, the models in the sensitivity analysis included the same predictors as in multivariable analysis with the addition of community‐based specialty pharmacy. See Table S4.

## 4 Discussion

In the largest study to date of persistence on PrEP medication, we observed 56% persistence from initiation to one year of follow‐up. For those on PrEP medication after the first year, we found 63% persistence in the year 2 period. Across the entire 2‐year span, only two out of every five users persisted on PrEP. Behaviour risk over time is not constant, so it is likely that some portion of individuals were no longer guidelines‐eligible for PrEP 22. But this is unlikely to be the only factor accounting for such high levels of PrEP cessation; such substantial behaviour modification to convert three‐fifths of individuals initiating PrEP to be no longer PrEP eligible is highly unlikely. Instead, factors that have previously been associated with PrEP cessation likely contributed: financial barriers, poor tolerance of medication side effects, changes in perceived risk, limited social or external support, and difficulty adhering to frequent provider and lab visits 11, 19, 23, 24, 25.

Previous studies identified high non‐persistence in the first year of PrEP medication, and data from the first year of follow‐up in the present study confirm this finding. Surprisingly, non‐persistence on PrEP medication was only moderately attenuated in the second year (37% non‐persistence in year 2, compared to 44% non‐persistence in year 1). This finding has substantial implications for PrEP retention programmes: sustained efforts are needed to retain PrEP users throughout their first two years of medication, and possibly for longer periods of time. Further research is needed to understand the nuances of patterns of entry, exit and reentry/reexit for PrEP medication.

Among our study population, the 18 to 24 age group had higher rates of non‐persistence on medication for all time periods. This finding is consistent with data on national PrEP prevalence trends 15, and with current PrEP prevalence data that find comparatively low prevalence of PrEP use for these groups 14. Despite this, 18‐ to 24‐year‐olds are a key group to target for retention on PrEP because they are among the groups at highest risk for transmission 25, 26. Younger individuals may be more likely to experience challenges in a number of areas, including cost navigation, fear of disclosure due to use of parental insurance, limited experience with the healthcare system, and financial barriers 25, 27. Long‐term persistence on PrEP medication for young people will be facilitated by no‐ or low‐cost access to services that are required for PrEP care, such as HIV testing/counselling and STI testing/treatment 28. Many of these services are available from community or non‐profit organizations, but the services of any particular clinic may not cover all tests (e.g. creatinine) and visit requirements (e.g. quarterly, with a clinician) for a PrEP prescription.

Consistent with previous PrEP studies 29, 30, 31, we found that men had higher odds of being persistent on PrEP medication over time. Female gender in a cohort study of Kaiser Permanente Northern California members was associated with discontinuation of PrEP over the three‐year study period (RR 2.6; 95% CI: 1.5 to 4.6) 30. Several studies have found that women underestimate their risk for acquiring HIV, 18, 32 a likely factor in PrEP non‐adherence and discontinuation. Additionally, women with PrEP indications may not be initiating PrEP; a study of PrEP uptake found that uptake among women was very low from 2010 to 2014, potentially highlighting a key barrier encountered by providers of identifying women with PrEP indications 33. Programmes and providers offering PrEP services should be aware of persistence disparities between men and women and seek to address concerns that may be of particular import for women.

Increased odds of persistence on PrEP medication were observed for having a copay of $20 or less, having commercial insurance, and attending a community‐based specialty pharmacy from the national chain. Lower copays have been associated with improved patient outcomes, including adherence and persistence in care, in numerous studies and for numerous health conditions 34, 35, 36. We found that individuals with a copay of $20 or less had slightly higher odds of persistence at one and two years of follow‐up than individuals with higher copays. To be included in this dataset, however, individuals must have made their first copayment and completed their first fill; persons who could not afford a high first copayment therefore never entered the dataset. Our findings are unable to address uninsured or underinsured individuals who did not initiate PrEP due to financial barriers. Given availability of pharmacy assistance programmes, medication copay may play a more minor role in persistence than payment for quarterly laboratory tests and office visits.

Primary payer was a significant predictor of persistence, with commercial individuals comprising the largest proportion and having the highest odds of persistence. There is a mixed consensus in the literature on the role of insurer for medication adherence and persistence, with some studies identifying lack of insurance coverage as a barrier 4, 20, 37, 38, others finding improved patient outcomes on commercial insurance compared to government insurance 39, 40, and one not finding a difference between persistence on medication for those with government insurance compared to those with commercial 41.

The present study has some strengths, including that the dataset represents a substantial proportion (over 7000) of the estimated 70,395 PrEP users active in the fourth quarter of 2017 in the United States 14. This study was also conducted using observational, real‐world data, which contributes towards filling a current gap in the PrEP literature.

There are a number of limitations to this study. Data are from a single pharmacy chain, and therefore individuals changing pharmacies could be persistent on PrEP but classified as non‐persistent. To understand the impact of this known bias, we conducted a sensitivity analysis among individuals that had filled at least one prescription subsequent to the end of the two‐year period of observation. We found little impact in terms of the magnitude of results and associations in the models. Individuals in the dataset could have initiated and discontinued PrEP prior to 2015 at a different pharmacy, although it is unclear in which direction this might introduce bias.

Another limitation is selection bias; a full assessment of the relative representativeness of this pharmacy chain nationally is outside the scope of this analysis. Because of the widespread coverage of the pharmacy chain in 49 states and the 20 most populous cities, the dataset at minimum incorporates data from many key areas of the United States. Regional variations of PrEP prescribing patterns and norms may be a factor in persistence, and future research should consider geographic region as a factor of interest.

Although it is not clinically recommended, persons may use TDF‐FTC for chronic Hepatitis B management and our dataset does not allow for exclusion of this group. In order to have been included in this analysis, an individual must have filled at least 60 days of TDF‐FTC. Therefore, individuals who were early discontinuers of PrEP due to side effects, cost, copay limitations or other challenges are undercounted, biasing our estimate of persistence. The measure of persistence we used was defined as at least 75% of months in a period; had a stricter definition been employed, such as requiring 90% of months in a period, persistence estimates would be lower. Individuals taking PrEP on an event‐based dosing or other irregular schedule may be misclassified as not persistent on PrEP medication. We do not anticipate the impact of this to be particularly high because current CDC guidance does not recommend this dosing schedule 10. Individuals who are recurrently taking post‐exposure prophylaxis (PEP) may be misclassified as non‐persistent on PrEP, although this is likely rare because monotherapy for PEP is uncommon.

Data on prescription copay have several limitations. First, only prescriptions sold were analysed, so prescriptions not filled due to high copay were not captured. Second, we analysed average monthly copay costs over the total length of time PrEP medication was filled and were therefore unable to detect if monthly differences in copay costs affected persistence.

A substantial limitation is that individuals who paid for PrEP using manufacturer's medication or copay assistance programmes were unable to be isolated as a separate category in the primary payer variable, and we were unable to quantify the true extent of their use or their impact on persistence. Due to privacy concerns, data were aggregated at the year level instead of being analysed on a monthly level, which may mask data trends. Lastly, because the data source is administrative in nature, many demographic variables are not collected. Thus, key variables known to correlate with PrEP uptake and persistence on medication, including race/ethnicity, income, sexual orientation and gender identity, were not available.

## 5 Conclusions

Using pharmacy refill data to measure persistence on PrEP medication over two years of follow‐up, we found substantial non‐persistence on PrEP medication in both year 1 and year 2. Across the entire 2‐year period, two out of every five users persisted on PrEP. Demographic, financial and pharmacy factors were associated with persistence. PrEP interventions targeted at increasing persistence are merited and should be conducted throughout at least the first two years of medication. Programmes should be aware of disparities in PrEP persistence, with young adults, women, and those not on commercial insurance more likely to not be persistent. Further research is needed to explore how social, structural or individual factors may undermine or enhance persistence on PrEP medication, and to develop and test interventions to assist persistence as indicated.

## Competing interests

KCC declares no competing interests. RJH, HSK and AD are employees of Walgreen Co. AJS is a Co‐Investigator on a grant from the Gilead Foundation.

## Authors’ contributions

KCC, AJS, HK, RH and AD designed and implemented the study. KCC did the statistical analyses with support from AJS, HK and RH. KCC and AJS drafted the manuscript. KCC, AJS, HK, RH and AD contributed to the interpretation and presentation of the findings. All authors approved the final version of this manuscript for submission.

## Supporting information


**Table S1.** Persistence on PrEP medication in year 1 (zero to twelve months), year 2 (twelve to twenty‐four months), and initiation to year 2 (zero to twenty‐four months) among individuals who initiated PrEP in the United States, 2015
**Table S2.** Sensitivity analysis of demographic characteristics of individuals who initiated HIV preexposure prophylaxis and filled any other prescription following their final PrEP fill in the United States, 2015 at initiation
**Table S3.** Sensitivity analysis of persistence on PrEP medication in year 1 (zero to twelve months), year 2 (twelve to twenty‐four months), and initiation to year 2 (zero to twenty‐four months) among individuals who initiated PrEP in the United States, 2015 for selected variables
**Table S4.** Sensitivity analysis of factors associated with persistence on PrEP medication in year 1 (zero to twelve months), year 2 (twelve to twenty‐four months), and initiation to year 2 (zero to twenty‐four months) among individuals who have filled a prescription other than PrEP following their final prep fill in the study period, 2015 to 2017
