# Supplementary material for: Persistence on HIV preexposure prophylaxis medication over a 2‐year period among a national sample of 7148 PrEP users, United States, 2015 to 2017
Source: J Int AIDS Soc. 2019 Feb 18;22(2):e25252. doi: 10.1002/jia2.25252 (PMC6378757; doi:10.1002/jia2.25252)
Supplement: Supplementary file 1 — Table S1. Persistence on PrEP medication in year 1 (zero to twelve months), year 2 (twelve to twenty‐four months), and initiation to year 2 (zero to twenty‐four months) among individuals who initiated PrEP in the United States, 2015 Table S2. Sensitivity analysis of demographic characteristics of individuals who initiated HIV preexposure prophylaxis and filled any other prescription following their final PrEP fill in the United States, 2015 at initiation Table S3. Sensitivity analysis of persistence on PrEP medication in year 1 (zero to twelve months), year 2 (twelve to twenty‐four months), and initiation to year 2 (zero to twenty‐four months) among individuals who initiated PrEP in the United States, 2015 for selected variables Table S4. Sensitivity analysis of factors associated with persistence on PrEP medication in year 1 (zero to twelve months), year 2 (twelve to twenty‐four months), and initiation to year 2 (zero to twenty‐four months) among individuals who have filled a prescription other than PrEP following their final prep fill in the study period, 2015 to 2017 [file JIA2-22-e25252-s001.docx]

**Supporting Information**

**Table S1: Persistence on PrEP Medication in Year 1 (0-12 months), Year 2 (12-24 months), and Initiation to Year 2 (0-24 months) Among Individuals Who Initiated PrEP in the United States, 2015**

|  | | **PrEP Initiation** | **Persistence in Year 1**  **(0-12 months)** | | **Persistence in Year 2**  **(13-24 months)** | | **Persistence from Initiation to Year 2 (0-24 months)** | |
| --- | --- | --- | --- | --- | --- | --- | --- | --- |
|  | | n | n | Percent Persistent | n | Percent Persistent | n | Percent Persistent |
| All | | 7,148 | 4,030 | 56% | 2,521 | 63% | 2,951 | 41% |
| Age | |  |  |  |  |  |  |  |
|  | 18-24 | 784 | 339 | 43% | 183 | 54% | 227 | 29% |
|  | 25-29 | 1,552 | 815 | 53% | 452 | 55% | 539 | 35% |
|  | 30-39 | 2,521 | 1,409 | 56% | 872 | 62% | 1,041 | 41% |
|  | 40-49 | 1,432 | 912 | 64% | 621 | 68% | 704 | 49% |
|  | 50+ | 855 | 553 | 65% | 392 | 71% | 439 | 51% |
| Gender | |  |  |  |  |  |  |  |
|  | Men | 6,900 | 3,944 | 57% | 2,479 | 63% | 2,901 | 42% |
|  | Women | 244 | 84 | 34% | 41 | 49% | 49 | 20% |
| Monthly average copay | |  |  |  |  |  |  |  |
|  | $20 or less | 5,531 | 3,220 | 58% | 2,052 | 64% | 2,387 | 43% |
|  | More than $20 | 1,614 | 808 | 50% | 468 | 59% | 562 | 35% |
| Payer (primary during entire period) | |  |  |  |  |  |  |  |
|  | Commercial | 5,699 | 3,366 | 59% | 2,165 | 64% | 2,514 | 44% |
|  | Government | 1,097 | 506 | 46% | 267 | 53% | 321 | 29% |
|  | Cash/Other | 352 | 158 | 45% | 89 | 56% | 116 | 33% |
| Pharmacy type | |  |  |  |  |  |  |  |
|  | Community-based specialty pharmacy | 1,057 | 673 | 64% | 442 | 65% | 512 | 48% |
|  | Traditional retail pharmacy | 6,091 | 3,357 | 55% | 2,079 | 62% | 2,439 | 44% |

Note: To be considered persistent, individuals must have had 16 days of medication available per calendar month for three-quarters of months in each interval. Only individuals persistent at 1 year of follow-up (months 0-12) were eligible to be considered persistent at 2 years (months 13-24). Primary payer reflects the source of payment used most frequently in the study period. Some data points are missing for up to 4 individuals.

**Table S2: Sensitivity Analysis of Demographic Characteristics of Individuals Who Initiated HIV Pre-Exposure Prophylaxis and Filled any Other Prescription Following their Final PrEP Fill in the United States, 2015 at Initiation**

|  | | | All | Individuals with any Post-PrEP Fill |
| --- | --- | --- | --- | --- |
|  | | | n (%) | n (%) |
| Total sample | | | 7,148 | 5,837 |
| Age | | |  |  |
|  | | 18-24 | 784 (11%) | 574 (10%) |
|  | | 25-29 | 1,552 (22%) | 1,197 (20%) |
|  | | 30-39 | 2,521 (35%) | 2,076 (36%) |
|  | | 40-49 | 1,432 (20%) | 1,238 (21%) |
|  | | 50+ | 855 (12%) | 748 (13%) |
| Gender | | |  |  |
|  | | Men | 6,900 (97%) | 5,640 (97%) |
|  | | Women | 244 (3%) | 193 (3%) |
| Monthly average copay | | |  |  |
|  | | $20 or less | 5,531 (77%) | 4,485 (77%) |
|  | | More than $20 | 1,614 (23%) | 1,349 (23%) |
|  | | Mean (SD) | 20 (78) | 20 (74) |
| Payer (primary during entire period) | | |  |  |
|  | | Commercial | 5,699 (80%) | 4,765 (81%) |
|  | | Government | 1,097 (15%) | 802 (14%) |
|  | | Cash/Other | 352 (5%) | 270 (5%) |
| Pharmacy type | | |  |  |
|  | Community-based specialty pharmacy | | 1,057 (15%) | 853 (15%) |
|  | Traditional retail pharmacy | | 6,091 (85%) | 4,984 (85%) |
| Distance to pharmacy from home (miles) | | |  |  |
|  | 0 to <1 miles | | 5,293 (74%) | 4,376 (75%) |
|  | 1 to < 2 miles | | 1,235 (17%) | 990 (17%) |
|  | 2+ miles | | 620 (9%) | 471 (8%) |
|  | Mean (SD) | | 1 (3) | 1 (2) |
| Urban/Rural Status | | |  |  |
|  | Urban | | 3,093 (43%) | 2,543 (44%) |
|  | Less dense urban | | 1,458 (20%) | 1,195 (21%) |
|  | Suburban | | 2,257 (32%) | 1,834 (30%) |
|  | Rural | | 340 (5%) | 265 (5%) |

**Table S3: Sensitivity Analysis of Persistence on PrEP Medication in Year 1 (0-12 months), Year 2 (12-24 months), and Initiation to Year 2 (0-24 months) Among Individuals Who Initiated PrEP in the United States, 2015 for Selected Variables**

|  | | **PrEP Initiation** | | **Persistence in Year 1**  **(0-12 months)** | | | | **Persistence in Year 2**  **(13-24 months)** | | | | **Persistence from Initiation to Year 2**  **(0-24 months)** | | | |
| --- | --- | --- | --- | --- | --- | --- | --- | --- | --- | --- | --- | --- | --- | --- | --- |
|  | | All | Individuals with any Post-PrEP Fill | All | | Individuals with any Post-PrEP Fill | | All | | Individuals with any Post-PrEP Fill | | All | | Individuals with any Post-PrEP Fill | |
|  | | n | n | n | Percent Persistent | n | Percent Persistent | n | Percent Persistent | n | Percent Persistent | n | Percent Persistent | n | Percent Persistent |
| All | | 7,148 | 5,837 | 4,030 | 56% | 3,500 | 60% | 2,521 | 63% | 2,386 | 68% | 2,951 | 41% | 2,746 | 47% |
| Age | |  |  |  |  |  |  |  |  |  |  |  |  |  |  |
|  | 18-24 | 784 | 574 | 339 | 43% | 274 | 48% | 183 | 54% | 170 | 62% | 227 | 29% | 203 | 35% |
|  | 25-29 | 1,552 | 1,197 | 815 | 53% | 667 | 56% | 452 | 55% | 417 | 63% | 539 | 35% | 484 | 40% |
|  | 30-39 | 2,521 | 2,076 | 1,409 | 56% | 1,230 | 59% | 872 | 62% | 823 | 67% | 1,041 | 41% | 971 | 47% |
|  | 40-49 | 1,432 | 1,238 | 912 | 64% | 826 | 67% | 621 | 68% | 601 | 73% | 704 | 49% | 673 | 54% |
|  | 50+ | 855 | 748 | 553 | 65% | 501 | 67% | 392 | 71% | 374 | 75% | 439 | 51% | 414 | 55% |
| Gender | |  |  |  |  |  |  |  |  |  |  |  |  |  |  |
|  | Men | 6,900 | 5,640 | 3,944 | 57% | 3,426 | 61% | 2,479 | 63% | 2,349 | 69% | 2,901 | 42% | 2,701 | 48% |
|  | Women | 244 | 193 | 84 | 34% | 72 | 37% | 41 | 49% | 36 | 50% | 49 | 20% | 44 | 23% |

Note: To be considered persistent, individuals must have had 16 days of medication available per calendar month for 9 months of months 0-12, 13-24, and 0-24, respectively. Only individuals persistent at 1 year of follow-up (months 0-12) were eligible to be considered persistent at 2 years (months 13-24).

**Table S4: Sensitivity Analysis of Factors Associated with Persistence on PrEP Medication in Year 1 (0-12 months), Year 2 (12-24 months), and Initiation to Year 2 (0-24 months) Among Individuals Who Have Filled A Prescription Other Than Prep Following Their Final Prep Fill in the Study Period, 2015-2017**

|  | | **Persistence in Year 1 (0-12 months)** | | **Persistence in Year 2 (13-24 months)** | | **Persistence from Initiation to Year 2 (0-24 months)** | |
| --- | --- | --- | --- | --- | --- | --- | --- |
|  | | n = 5,830 | | n = 3,496 | | n = 5,830 | |
|  | | Bivariate  OR (95% CI) | Multivariable aOR (95% CI) | Bivariate  OR (95% CI) | Multivariable aOR (95% CI) | Bivariate  OR (95% CI) | Multivariable aOR (95% CI) |
| Age | |  |  |  |  |  |  |
|  | 18-24 | Ref | Ref | Ref | Ref | Ref | Ref |
|  | 25-29 | 1.38 (1.12, 1.68) | 1.34 (1.09, 1.64) | 1.02 (0.76, 1.36) | 1.03 (0.77, 1.38) | 1.24 (1.01, 1.53) | 1.20 (0.97, 1.48) |
|  | 30-39 | 1.59 (1.32, 1.92) | 1.57 (1.30, 1.90) | 1.24 (0.94, 1.62) | 1.26 (0.96, 1.66) | 1.61 (1.33, 1.95) | 1.59 (1.31, 1.93) |
|  | 40-49 | 2.20 (1.79, 2.69) | 2.23 (1.81, 2.73) | 1.63 (1.23, 2.18) | 1.72 (1.29, 2.30) | 2.18 (1.77, 2.67) | 2.22 (1.80, 2.73) |
|  | 50+ | 2.22 (1.78, 2.78) | 2.33 (1.86, 2.93) | 1.80 (1.31, 2.47) | 1.99 (1.45, 2.74) | 2.27 (1.81, 2.83) | 2.40 (1.91, 3.02) |
| Gender | |  |  |  |  |  |  |
|  | Female | Ref | Ref | Ref | Ref | Ref | Ref |
|  | Male | 2.60 (1.93, 3.50) | 2.27 (1.67, 3.09) | 2.18 (1.37, 3.48) | 2.13 (1.32, 3.43) | 3.11 (2.21, 4.37) | 2.68 (1.88, 3.80) |
| Monthly average copay | |  |  |  |  |  |  |
|  | $20 or less | Ref | Ref | Ref | Ref | Ref | Ref |
|  | More than $20 | 0.68 (0.61, 0.77) | 0.62 (0.54, 0.70) | 0.72 (0.60, 0.85) | 0.67 (0.56, 0.80) | 0.65 (0.57, 0.73) | 0.59 (0.52, 0.67) |
| Payer (primary during entire period) | |  |  |  |  |  |  |
|  | Commercial | Ref | Ref | Ref | Ref | Ref | Ref |
|  | Government | 0.62 (0.53, 0.72) | 0.61 (0.52, 0.71) | 0.74 (0.60, 0.92) | 0.70 (0.56, 0.87) | 0.58 (0.50, 0.68) | 0.57 (0.48, 0.67) |
|  | Cash/Other | 0.57 (0.45, 0.73) | 0.56 (0.43, 0.72) | 0.63 (0.44, 0.90) | 0.62 (0.43, 0.89) | 0.62 (0.48, 0.80) | 0.60 (0.46, 0.78) |
| Pharmacy type | |  |  |  |  |  |  |
|  | Traditional retail pharmacy | Ref | Ref | Ref | Ref | Ref | Ref |
|  | Community-based specialty pharmacy | 1.49 (1.28, 1.74) | 1.46 (1.25, 1.72) | 1.35 (1.11, 1.65) | 1.33 (1.08, 1.63) | 1.59 (1.37, 1.84) | 1.56 (1.34, 1.82) |
| Distance to pharmacy from home (miles) | | NS |  | NS |  | NS |  |
| Urban/Rural Status | | NS |  | NS |  | NS |  |

NS=not significant at the p < 0.05 level
Note: To be considered persistent, individuals must have had 16 days of medication available per calendar month for three-quarters of months in each interval. Only individuals persistent at 1 year of follow-up (months 0-12) were eligible to be considered persistent at 2 years (months 13-24). Some data points are missing for some individuals.
